# Extended disease resistance emerging from the faecal nest of a subterranean termite

**DOI:** 10.1098/rspb.2013.1885

**Published:** 2013-11-07

**Authors:** Thomas Chouvenc, Caroline A. Efstathion, Monica L. Elliott, Nan-Yao Su

**Affiliations:** 1 Department of Entomology and Nematology, University of Florida, Institute of Food and Agricultural Sciences, 3205 College Avenue, Fort Lauderdale, FL 33314, USA; 2 Department of Plant Pathology, Fort Lauderdale Research and Education Center, University of Florida, Institute of Food and Agricultural Sciences, 3205 College Avenue, Fort Lauderdale, FL 33314, USA

**Keywords:** termites, symbiosis, Actinobacteria, nesting behaviour, entomopathogens

## Abstract

Social insects nesting in soil environments are in constant contact with entomopathogens but have evolved a range of defence mechanisms, resulting in both individual and social immunity that reduce the chance for epizootics in the colony, as in the case of subterranean termites. *Coptotermes formosanus* uses its faeces as building material for its nest structure that result into a ‘carton material’, and here, we report that the faecal nest supports the growth of Actinobacteria which provide another level of protection to the social group against entomopathogens. A *Streptomyces* species with *in vivo* antimicrobial activity against fungal entomopathogens was isolated from the nest material of multiple termite colonies. Termite groups were exposed to *Metarhizium anisopliae*, a fungal entomopathogen, during their foraging activity and the presence of *Streptomyces* within the nest structure provided a significant survival benefit to the termites. Therefore, this report describes a non-nutritional exosymbiosis in a termite, in the form of a defensive mutualism which has emerged from the use of faecal material in the nesting structure of *Coptotermes*. The association with an Actinobacteria community in the termite faecal material provides an extended disease resistance to the termite group as another level of defence, in addition to their individual and social immunity.

## 1. Introduction

**Figure 1.**
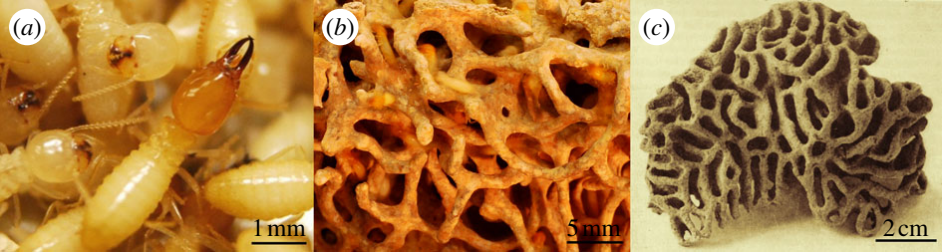
(*a*) *Coptotermes formosanus*, (*b*) carton material from *C. formosanus*, (*c*) *Termitomyces* fungal comb from *Macrotermes bellicosus* (modified from [2]). (Online version in colour.)

The Formosan subterranean termite, *Coptotermes formosanus*, builds underground nests with extensive foraging galleries for up to 150 m; a colony can contain more than 1 million individuals and it is considered as an important urban pest because of the damage it can do to wood structures [1]. While the inner lining of foraging galleries are coated with a termite faecal envelop, the voids created at foraging sites and the core of the nest are filled with a sponge-like structure composed of chewed wood particles mixed with faecal material, usually referred to as ‘carton material’ (figure 1). It was suggested to have first emerged in a common ancestor of *Coptotermes* and *Macrotermes* as a result of subterranean nest-building behaviour [3]. This building behaviour reinforces the nest structure and helps to maintain homoeostatic conditions inside the nest by reducing the temperature and moisture fluctuations from the surrounding soils [4] and the faecal envelop can be the host of a diverse microbial community [5]. By maintaining relatively high temperatures and humidity inside the nest, it was suggested that termites, as social insects living in highly dense colonies, would face an increased risk of epizootic events within the nest [6]. However, subterranean termites have evolved a range of disease resistance mechanisms, preventing harmful pathogens from spreading within the group [7,8]. Such mechanisms involve the individual cellular and humoral immune defence of each termite [9,10] but also includes hygienic behaviours which results in a social immunity [11–13]. In addition to the collective effort of the colony to limit the risk of epizootics of fungal entomopathogens, for example *Metarhizium anisopliae* [14], endogenous termite secretions may help to create a termite nesting environment poorly conducive for such fungi [15–19]. However, the description of the termite–*Metarhizium* relationship as a simple bipartite association appeared reductive [20]. We therefore propose that an exogenous protection may complete the termite's defence repertoire, here provided by a mutualistic association within the termite nest.

**Figure 2.**
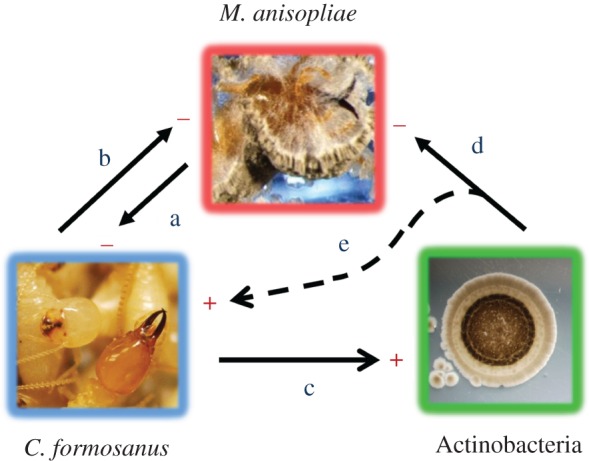
Interaction among *C. formosanus*, *M. anisopliae* and Actinobacteria inside the termite nest. (a) *Metarhizium anisopliae* is a virulent pathogen on individual termites [38], (b) termites have evolved disease resistance mechanisms that reduce the survival of entomopathogenous fungi inside the nest [7,8,19], (c) the termite faecal material from the carton material promotes the growth of some Actinobacteria (this study), (d) the Actinobacteria reduces the survival of *M. anisopliae* inside the termite nest (this study) and (e) by reducing the pressure from *M. anisopliae*, the Actinobacteria increases the chance of survival of *C. formosanus* (this study). (Online version in colour.)

Defensive mutualism involving insects and Actinobacteria have been extensively described [21–23]. Some ants [24–29], wasps [30–32] and beetles [33,34] are associated with Actinobacteria that are using the insect environment or the insect itself as a microniche, and can provide direct or indirect benefits for the insect against parasites or pathogens. Because the niche of such insects can be so unique, some of the Actinobacteria associated with these insects can be uncommon and may represent an untapped source for undiscovered antibiotics [32,35]. In subterranean termites, the carton material and the faecal lining in the foraging galleries provides a niche for the colonization of microorganisms [5,36] and specific Actinobacteria can provide secondary metabolites that benefit the termites against common soil entomopathogens. However, it can be difficult to provide evidence on the nature of an association between termites and microorganisms [20,37]. Therefore, to properly demonstrate a case of mutualism (see the electronic supplementary material, discussion), we established in this study the interaction of each organism in relation to each other (termites, fungal entomopathogens, Actinobacteria; figure 2), tested with *in vivo* conditions to confirm the reciprocal benefits that both the termites and Actinobacteria can obtain from this association.

## 2. Material and methods

### (a) Actinobacteria isolation from termite nest material

Carton material of five *C. formosanus* field colonies were collected from ‘bucket traps’ as described by Su & Scheffrahn [39]. Bucket traps were installed at active *C. formosanus* foraging sites (Broward County, FL, USA) and an autoclaved bundle of spruce wood was placed in the bucket. After two weeks in the ground, the partially consumed bundles of wood with voids filled with carton material were retrieved. Carton material samples were then collected under sterile conditions in the laboratory. To isolate Actinobacteria from termite carton material that would preferentially be associated with such a microniche, we followed the suggestions of Kaeberlein *et al.* [40] that poorly cultivable microorganisms may easily grow in culture if provided with the nutritional components of their natural environment, and of Shlatter *et al.* [41] that soil amended with cellulose and lignin can host higher densities of *Streptomyces* than soil poor in such resources. The termite faecal material integrated in the carton material is essentially composed of partially degraded plant material and may provide a natural selective media for termite associated Actinobacteria. We therefore used termite carton material as a nutritional base for a novel selective medium, termite faecal agar (TFA). It was prepared by adding 60 g of ground carton material from a *C. formosanus* laboratory nest to 1 l of water agar and then autoclaved. The use of a laboratory termite colony allowed us to obtain large quantities of carton material with homogeneous quality, which were necessary to prepare enough media for this large isolation protocol. To prevent the growth of fast growing fungi, 100 mg of cycloheximide per litre of agar was added to the TFA after sterilization. For each termite colony of origin, three subsamples of fresh carton material were subjected to serial dilutions (10^–4^ to 10^−6^) using deionized water, which were plated on the TFA medium (four replicates per dilution), and incubated in the dark at 28°C for 9 days.

Bacterial colony forming units (CFU) with an Actinobacteria morphotype were individually isolated and subcultured on 1/5 strength potato dextrose agar (1/5 PDA). In order to prevent a systematic bias in the isolation process, half of the replicates of the dilution plates were processed by T.C., whereas the other half was processed by C.A.E. Pure Actinobacteria colonies (500 + isolates) were archived in storage and a subset of representatives (based on morphology and antibiotic activity profile described below) from each nest of origin was selected for sequencing. The 16SrDNA sequence (1200 bp) was obtained by using the microbial sequencing service provided by Laragen (Culver City, CA, USA). 16SrDNA sequences were edited using DNA Baser v. 2.9 (Heracle software). Sequence alignment and phylogeny trees were computed with Mega v. 5.0, using neighbour-joining method with default parameters. Sequences were deposited in GenBank database under the accession numbers KC111822-1873.

### (b) Antimicrobial assay

Actinobacteria isolates obtained from *C. formosanus* carton materials were tested for their antimicrobial activity against two fungal entomopathogens, *M. anisopliae* (ATCC 98094) and *Beauveria bassiana* (undescribed isolate). In addition, isolates were tested against a range of microorganisms to determine a basic profile of their overall antimicrobial activity. These microorganisms represent species of Gram-negative and Gram-positive bacteria, as well as yeast and other fungi. Test species included *Bacillus subtilis* (ATCC 6633), *Serratia marcescens* (ATCC 43862) *Staphylococcus aureus* (NCIMB 9518), *Escherichia coli* (ATCC 25922), *Pseudomonas aeruginosa* (ATCC 25619), *Xantophomonas maltophilia* (ATCC 13636), *Klebsiella pneumonia* (BAA-2146), *Aspergillus nomius* (AsFL-07), *Penicillum* sp. (undescribed isolate) and *Saccharomyces cerevisiae* (ATCC 2601). Some of these microbial species are infectious to immune-compromised individuals in humans and are known to be problematic in hospitals as nosocomial diseases, owing to their acquired drug resistance to commonly used antibiotics [42]. Therefore, this preliminary screening not only aimed to provide a basic antimicrobial profile, but also to prospect for novel antibiotic agent.

The antimicrobial screening was accomplished by growing Actinobacteria isolates individually on 1/5 PDA, centred in the plate, for 4 days to allow diffusion of secondary metabolites through the plate (two replicates per isolate). The culture was then overlaid with 10 ml of water agar containing a concentrated cell or conidia suspension of the test organism (100 #CFU per ml). The incubation for a given test organism was done at their optimal growth temperatures [43] and measurements of the growth inhibition were done 24 h after overlay for bacteria and yeasts, and 72 h after overlay for fungi. The zone of growth inhibition was established by measuring the average distance (four orthogonal measurements) between the edge of the Actinobacteria colony and the edge of the zone of inhibition. Zones more than 40 mm indicated that the inhibition was complete over the entire plate. We are aware that testing for antimicrobial activity only in a 1/5 PDA medium (pH = 5.25) is a reductive approach as some Actinobacteria may require a different nutritional or pH environment to produce particular antimicrobial secondary metabolites, however, in this study, we restricted the screening for practical purpose and to focus our effort on a thorough termite bioassay.

### (c) *Metarhizium anisopliae* preparation

Because a vast majority of virulence studies and defence mechanisms studies in termites were performed with *M. anisopliae* as a model entomopathogen [7,8,37,38], this fungus species was selected as a representative for further *in vitro* and *in vivo* bioassays. Conidia of *M. anisopliae* were spread on 1/5 PDA and incubated at 28°C in the dark. After 48 h, single conidia colonies were transferred to 1/5 PDA containing one worker termite, previously killed and surface-sterilized for 1 h in a vial saturated with chloroform vapours [44]. The use of the sterilized termite is to provide proper nutrition for the fungus, which helps maintain its virulence through subculture. Inoculated plates were then incubated at 28°C for 14 days in the dark. Fresh conidia were harvested from these plates with a 0.05% Tween80 solution (for conidia suspension) and a stock suspension of 5 × 10^7^ viable conidia per millilitre was prepared (determined by conidia germination on 1/5 PDA). Conidia stock suspensions were stored at 4°C and used for dilutions (with sterile deionized water) within 15 days of the experiments described herein (97% viable conidia after 15 days).

### (d) *Streptomyces* #2338 as a representative

The isolate *Streptomyces* #2338 was used as a representative of a ribotype sampled from all five termite nests (see electronic supplementary material, figure S1). This ribotype also showed strong *in vitro* antifungal activity against the entomopathogenic fungus *M. anisopliae* and it was considered as a potential candidate for mutualism in *C. formosanus*. However, the observation of such antibiotic activity in a highly artificial environment may have little relevance biologically [45] and may not represent the antimicrobial activity that the isolate could display in a termite nest environment. Therefore, we first tested the interaction of *Streptomyces* #2338 and *M. anisopliae* in a termite nest-like environment, that is in the presence of termite carton material, before proceeding to the termite bioassay. A *Streptomyces* #2338 stock solution of 10^9^ viable cells per millilitre was obtained from 1/5 PDA subcultures and suspended with a 0.05% Tween80 solution.

### (e) Interaction between *Metarhizium anisopliae* and *Streptomyces* #2338 in a termite nest-like nutritional environment

Acetone-washed and triple-rinsed white sand (150–500 µm particle size) was autoclaved and used as a neutral soil matrix for all experiments. Two types of soil environment were tested on microbial growth: pure sand (oligotrophic, as a negative control), and sand amended with sterile termite carton material (75% sand, 25% carton). For each treatment, 10 g of sand or sand–carton mix were placed in a 10 cm sterile glass Petri dish and moistened with 2 ml of sterile deionized water. There were four treatments per type of soil (three replicates per treatment): control treatment (deionized water), *Streptomyces* #2338 only (10^6^ cell per gram of dry soil), *M. anisopliae* (10^5^ conidia per gram of dry soil) and *Streptomyces* #2338 + *M. anisopliae* (concentrations as above). Microbial growth was monitored every 7 days for 42 days (except at 35 days) using the following protocol: for each Petri dish, three subsamples of 0.6 g (0.5 g dry weight equivalent) were subjected to serial dilutions (three replicate for each subsamples) and plated on 1/5 PDA for all treatments from both soil environments to count for CFUs. However, for the *Streptomyces* #2338 + *M. anisopliae* treatments, the serial dilutions of the subsamples were replicated and plated separately on 1/5 PDA amended with cycloheximide (100 mg l^−1^) to monitor only *Streptomyces* #2338 growth, and on 1/5 PDA amended with chloramphenicol (100 mg l^−1^) to monitor only *M. anisopliae* growth. CFUs were counted 3 days after plating the serial dilutions (*n* = 27 per treatment, per dilution).

### (f) Inhibition of *Metarhizium anisopliae* by *Streptomyces* #2338: exclusion and/or chemical interference?

After the inhibition of *M. anisopliae* by *Streptomyces* #2338 was determined in a termite nest-like environment, the type of inhibition between both microorganisms was investigated. In a subterranean termite faecal niche, the observed inhibition can be owing to three possible mechanisms: (i) exclusion, where *Streptomyces* #2338 outcompetes *M. anisopliae* by dominating the niche with a high density, to reduce the access to nutrients by *M. anisopliae*, (ii) chemical interference, where *Streptomyces* #2338 produces antifungal secondary metabolites that reduce the growth of *M. anisopliae* and (iii) a combination of both mechanisms. To test the origin of the inhibition, an identical protocol as above (interaction in a termite nest-like environment) was repeated, but instead of using *Streptomyces* #2338, the isolate *Streptomyces* #2345 was used. This isolate was used for comparison with #2338 because they were both isolated from the same termite carton material, both belong to the same 16S subclade, but contrary to #2338, the isolate #2345 did not display any antibiotic activity *in vitro* (see electronic supplementary material, figure S1). If the growth of isolate #2345 was similar to #2338 in a termite faecal environment, without affecting the growth of *M. anisopliae*, then according to the *in vitro* assay, this would suggest that the inhibition of *M. anisopliae* by #2338 in a carton material environment was owing to chemical interference.

### (g) Termite preparation for the arena bioassay

Termites were collected from five field colonies of the Formosan subterranean termite *C. formosanus* in Broward County, FL, USA and two colonies were collected in New Orleans, LA, USA using the ‘bucket trap’ method [39] processed in the laboratory according to Tamashiro *et al.* [46] and kept in groups of at least 2000 for 10–15 days in containers at 28°C before use in the bioassay. For each colony, groups of 50 termites (45 workers + 5 soldiers) were prepared. The groups of termites were then introduced into a planar arena (described below). In our study, the planar arena has the advantage over other standard protocols (Petri dish, jar) because it provides a soil environment enabling termites to forage and establish their own tunnel structure, while allowing continuous monitoring. The advantages of the use of this protocol have been fully discussed [47]. For each of the four microbial treatments discussed below, 14 replicates (two per termite colony per microbial treatment) were prepared, for a total of 56 arenas (2800 termites).

### (h) Soil treatments for the arena bioassay

Four soil treatments were prepared for the arena bioassay (table 1). For each arena, 15 g of sterile sand (dry weight) was placed in a sterile glass Petri dish, and mixed with 2.6 ml of a 15% sterile termite carton material in water suspension. This suspension aims to provide enough nutrients for microorganisms to establish a stable density and to express their metabolism as in a field termite nest. This carton material treatment of the sand likely approximates a termite gallery environment in which faecal deposition is present. For the *Streptomyces* #2338 only treatment and mixture of *Streptomyces* #2338 plus *M. anisopliae* treatment, 0.2 ml of 0.05% Tween80 solution containing *Streptomyces* #2338 was added to the wet sand–carton suspension in the Petri plates. This treatment simulates a termite gallery with the Actinobacteria community already established. An equivalent amount (0.2 ml) of 0.05% Tween80 solution was added to the control treatment Petri plates (no *Streptomyces* and no *M. anisopliae*) and to the future *M. anisopliae* treatment Petri plates. The Petri plates with the mixtures were kept at 28°C in the dark for 15 days. Finally, after 15 days of incubation, depending on the treatment, Petri dishes received 0.2 ml of a 0.05% Tween80 solution or a *M. anisopliae* conidia suspension in 0.05% Tween80 solution. Table 1 summarizes the soil microbial treatments. The control treatment contains only the sand, termite carton nest material water suspension and Tween80 solution.

**Table1.** Preparation of the sand matrix prior to introduction in the arena (15 g of sterile dry sand per arena, 14 replicates per treatment. The groups of 50 termites (45 workers + 5 soldiers) were introduced within 2 h after the last treatment and their survivorship was monitored daily for 60 days after introduction).

sand treatment	day 0	day 15
control	2.6 ml carton suspension + 0.2 ml 0.05% Tween80	0.2 ml 0.05% Tween80
*Streptomyces* #2338	2.6 ml carton suspension + 0.2 ml *Streptomyces* #2338 suspension (10^5^ cells per gram of dry sand)	0.2 ml 0.05% Tween80
*Metarhizium anisopliae*	2.6 ml carton suspension + 0.2 ml 0.05% Tween80	0.2 ml of *M. anisopliae* suspension (10^4^ conidia per gram of dry sand)
*Streptomyces* #2338 + *M. anisopliae*	2.6 ml carton suspension + 0.2 ml *Streptomyces* #2338 suspension (10^5^ cells per gram of dry sand)	0.2 ml of *M. anisopliae* suspension (10^4^ conidia per gram of dry sand)

Suspension densities of *M. anisopliae* and *Streptomyces* #2338 were previously adjusted by dilution in 0.05% Tween80 to obtain the final concentrations. Expected densities of viable conidia and cells per gram of soil were confirmed by plating serial dilutions of 0.6 g of soil samples from each preparation on 1/5 PDA and counting CFUs after 3 days, with a ±5% margin of error. Within 2 h after the 0.2 ml of Tween80 control solution or *M. anisopliae* suspension was added, the final sand mixture treatments (15 g dry sand + 3 ml of Tween80, with or without microbial suspensions) were introduced into individual planar arenas as described below. In this study, the logic behind establishing the *Streptomyces* population in the sand–carton mixture for 15 days prior to introducing termites was to simulate a termite tunnel condition where *Streptomyces* had the opportunity to be established and produce its secondary metabolites within its environment. *Metarhizium anisopliae* was inoculated just prior to the termite introduction to simulate the encountering of the fungus by the termites during foraging in the soil.

### (i) Arena bioassay set-up

Planar arenas (see electronic supplementary material, figure S2) as described in Chouvenc *et al.* [47] were composed of two sheets of transparent Plexiglas (12 × 12 × 0.2 cm in thickness) separated from each other by Plexiglas laminates (2 cm in width and 0.2 cm in thickness) on the four sides, creating a 10 × 10 × 0.2 cm space inside the arena. A 0.8 × 0.8 × 0.2 cm spacer was placed in the centre of the arena. The two sheets of Plexiglas and the central spacer were held together by a 3 mm-diameter screw, in order to maintain the 0.2 cm space layer evenly throughout the entire arena. A 0.4 mm diameter hole was drilled on the top Plexiglas sheet, 2 cm away from the spacer–screw hole, for adding liquids with the help of a syringe and to allow a small amount of air flow. A 5-mm-diameter hole was provided in one corner for the introduction of the termites into the arena chamber. Before assembly, all elements were washed with soap, immersed in bleach (3% sodium hypochlorite solution) for 2 h and rinsed three times with sterile deionized water. A sterile cellulose absorbent pad (45 mm in diameter, 2 mm thick) was placed below the central spacer, centred with the 0.4 mm hole. The arena was filled with 18 g of wet sand–carton (15 g of sterile dry sand, 150–500 µm size and 3 ml of treatment solution, see ‘soil treatment’ above), leaving a band of 10 by 2.5 cm empty on one border, exposing the cellulose absorbent pad to the empty space, which allow termites to find a food source immediately. The arena pieces were held together with eight 1-cm binder clips and the mounted arena was set horizontally. The four sides of the arena were sealed by hot glue, in order to prevent sand desiccation. One ml of sterile deionized water was injected via the 0.4 mm centre hole onto the absorbent pad. A group of 50 termites (45 workers + 5 soldiers) was introduced into the arena with the help of a small funnel, and the introduction hole was sealed with a thin transparent plastic cover after all termites entered the arena. All 56 arenas were placed at 28°C in the dark and termite mortality was monitored daily for 60 days by taking digital pictures with brief illumination from beneath with LED lights. Dead termites (determined by their lack of movement and absence of reaction when in contact with nest-mates) were not removed as they were buried or cannibalized. During the daily census, cannibalized and buried cadavers were counted as ‘dead’ in this study, as this aspect was previously discussed in [14].

### (j) Statistical analysis

The effect of the presence of a microorganism on the growth of another microorganism in sand–carton material mixture was established by comparing the number of CFUs at 42 days in the two treatments (presence or absence of a competitor) with a Student *t*-test. Because of the growth rates of the *Streptomyces* isolates, the data were log-transformed. The absence of growth of microorganisms over time in plain sand was confirmed by the absence of positive correlation between time and number of CFUs (Pearson correlation).

A Cox proportional-hazard regression analysis (using the program R-Project for statistical computing, v. 2.4; http//cran.r-project.org/) was performed on all individual termites and a Wald statistic was generated. The factors tested were: colony of origin, fungal treatment and Actinobacteria treatment. The resulting hazard function defines the instantaneous rate of death at a particular time. Pairwise comparisons of the hazard ratio of death among treatments were adjusted by the Holm–Bonferroni method (*α* = 0.05).

## 3. Results and discussion

### (a) Isolation of Actinobacteria from termite nest materials

More than 500 Actinobacteria isolates were obtained from five *C. formosanus* colonies, 37 ribotypes (16S rDNA) were identified and 70% of isolates showed *in vitro* antimicrobial activity against a range of Gram-positive and Gram-negative bacteria, yeast and fungi (see electronic supplementary material, figure S1). *Streptomyces* isolates were the most common Actinobacteria genus found in all termite nest materials (62%), but species of *Kitasatospora*, *Pseudonocardia*, *Nocardioides*, *Nocardia*, *Gordonia*, *Mycobacterium* and *Kribbella* were also isolated. A particular *Streptomyces* 16S ribotype was found in the nest material of all five termite colonies at 10^6^ cells and higher per gram of carton material (all isolates were obtained from the 10^−6^ serial dilutions), and was the most bioactive isolate against entomopathogenic fungi such as *M. anisopliae* and *B. bassiana in vitro*. After this preliminary antimicrobial screening, we used *Streptomyces* #2338 isolate as a representative to determine whether an *in vivo* mutualistic relationship could be established between termites and this *Streptomyces* isolate.

### (b) *In vivo* interaction between *Streptomyces* and *Metarhizium anisopliae*

First, we determined that termite carton material (after sterilization) provides proper nutritional requirements to maintain a stable population of *Streptomyces* #2338 within the termite nest (see electronic supplementary material, figure S3*a*). When *M. anisopliae* was introduced into the carton material with *Streptomyces* #2338, the *Streptomyces* growth was not affected (*t*-test, *n* = 54, *p* = 0.76). Second, *M. anisopliae* was able to thrive when alone in the carton material, but not when associated with *Streptomyces* #2338 (see electronic supplementary material, figure S3*b*) as the fungal growth was reduced by 69% after six weeks (*t*-test, *n* = 54, *p* < 0.001). Therefore, in a termite carton material nutritional environment, *Streptomyces* #2338 had a direct negative impact on the growth of *M. anisopliae*. Third, to determine whether the growth reduction of *M. anisopliae* in a carton environment was the result of exclusion competition or chemical interference [48] by *Streptomyces* #2338, a different isolate of *Streptomyces* (#2345) with no *in vitro* antifungal activity was used in an identical protocol. We found that *Streptomyces* #2345 grew in a carton material environment as previously observed with *Streptomyces* #2338, but that *M. anisopliae* growth was not affected by the presence of *Streptomyces* #2345 (see electronic supplementary material, figure S4). This result supports the hypothesis that the inhibition of *M. anisopliae* by *Streptomyces* #2338 in a nutritional environment similar to the termite nest is by chemical interference, with the production of antimicrobial compounds.

### (c) Effect of *Streptomyces* on *Coptotermes formosanus* survival

**Figure 3.**
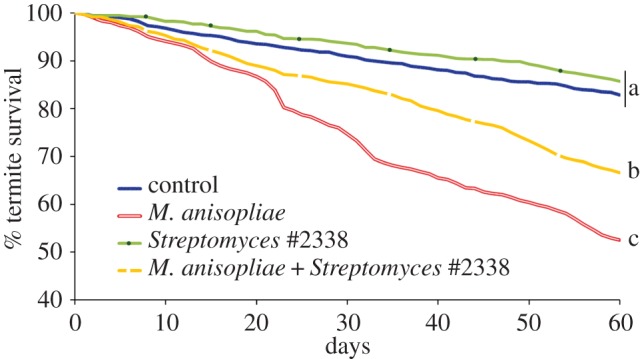
Daily termite survival over 60 days in planar arenas (mini-nests [47]). The arenas contained sand treated with a sterile termite carton material suspension, and four different treatments: control, *Streptomyces* only, *M. anisopliae* only, *Streptomyces* + *M. anisopliae* (14 replicates per treatment). Different letters among treatments indicate a significant difference in their hazard ratio of death (Cox regression, pairwise comparison adjusted by the Holm–Bonferroni method (*α* = 0.05)). (Online version in colour.)

The direct impact of *Streptomyces* #2338 on termite survival when exposed to entomopathogens remained to be determined. We used termite ‘mini-nests’ to maintain groups of termites in their own gallery system for extended time [47]. Groups of 50 termites foraged in the mini-nest for 60 days, where the sterile sand–carton material in the mini-nest was preliminarily treated with a combination of microorganisms and their survivorship was recorded daily (figure 3). The termite colony of origin was not a significant predictor of survival but both *Streptomyces* #2338 and *M. anisopliae* were independent predictors of termite survival (0.675 and 3.03 hazard ratios of death, respectively, Wald = 221.6, d.f. = 2, *p* < 0.001). Termite groups that were able to forage and establish their tunnel system in sand–carton material already containing *Streptomyces* #2338 had similar survival to the control groups (0.81 times the hazard ratio of death of the controls, Wald = 2.39, d.f. = 1, *p* = 0.12). Such results implied that *Streptomyces* #2338 had no virulence toward the termites when incorporated within the tunnel walls. Groups of termites that were exposed to *M. anisopliae* showed a significantly lower survival than the control groups (3.37 times the hazard ratio of death, Wald = 3.4, d.f. = 1, *p* < 0.001), confirming the virulence of the fungus on termites at the given density (10^4^ conidia g^−1^, ≈LD50 at 60 days [14]) in their foraging environment. Finally, termite groups that were kept in mini-nests treated with both *Streptomyces* #2338 and *M. anisopliae*, had higher survival than the groups treated with *M. anisopliae* only (0.63 times the hazard ratio of death, Wald = 29.8, d.f. = 1, *p* < 0.001). Therefore, the presence of *Streptomyces* #2338 in the termite galleries, once established in the termite faecal material, provided direct protection to the termites against a soil entomopathogenic fungus.

### (d) Extended disease resistance

**Figure 4.**
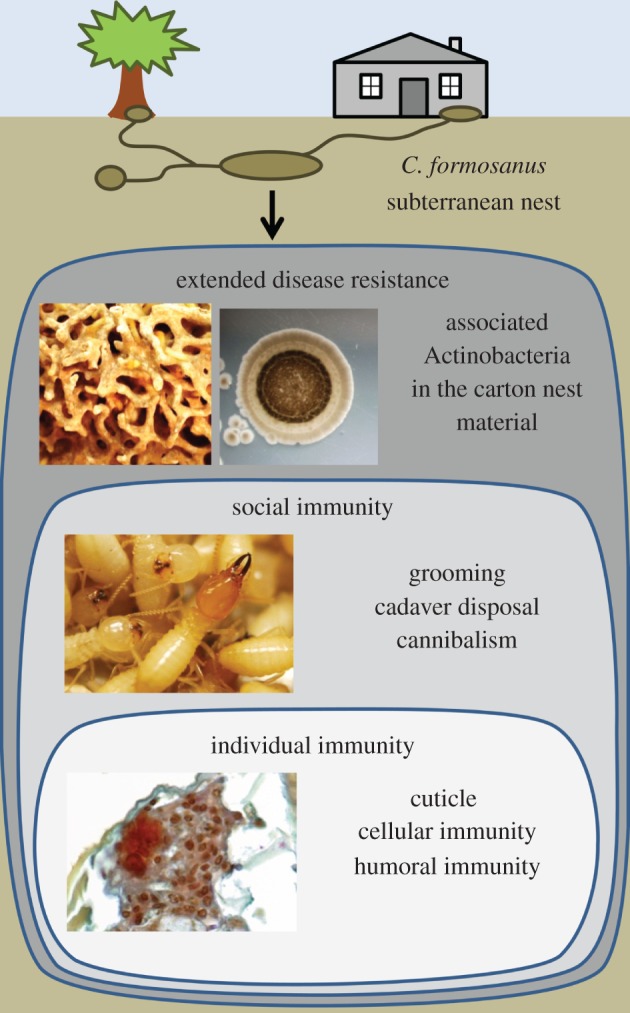
Different levels of immunity in *C. formosanus*. Each termite possesses an ‘individual immunity’ against pathogens with a physical defence (cuticle) and physiological defence (humoral and cellular immunity). As a group (superorganism), termite interactions, hygienic behaviours and secretions provide a ‘social immunity’. As a by-product of their building behaviour, the termite carton nest material and the faecal lining of the gallery walls provide a physical separation against surrounding soil entomopathogens, and also provide a niche to a community of beneficial Actinobacteria that reduces the chance for entomopathogens to invade the nest structure (extended disease resistance). (Online version in colour.)

Our study demonstrated that the presence of *Streptomyces* #2338 in the termite carton material reduced the pathogenic pressure from *M. anisopliae* against *C. formosanus* and represents a case of defensive mutualism. It was previously established that subterranean termites and other termites have evolved a range of defence mechanisms which resulted in an efficient interaction of individual and social immunity, reducing the chances of fungal epizootics in the colony [7,8,14,19,44]. We report here that the association of termites with beneficial microorganisms within their nest material provides additional protection to termites against the risks of epizootic, which may help to explain the repeated failures of biological control attempts against subterranean termites [37]. A analogous beneficial association was recently described in ants [49,50]; although in such cases, the mutualistic Actinobacteria forms a protective biofilm on the surface of specialized structures of the ants’ cuticle. In the absence of such structures in termites, an analogue association emerged from the termites nest structure, considered the extended phenotype of the colony [47,51]. The overall termite nest environment is coated with a layer of faecal-based material [4], which favours the growth of potentially beneficial Actinobacteria, for instance *Streptomyces* #2338, and reduces the pressure on termites from soil pathogens as an extended disease resistance (figure 4), which could be part of an extended immunity.

### (e) Origin and nature of the mutualism

Termites (Isoptera) are successful lignocellulose decomposers owing to the acquisition of diverse nutritional mutualists through their evolutionary radiation [52]. The transition between lower and higher termites (Coptotermitinae–Macrotermitinae) represents a key change in the biology of termites as the digestion process moved from an internal rumen with mutualistic protozoans, to an external rumen with the mutualistic fungus, *Termitomyces* [53]. This transition involved the loss of protozoans in all higher termites, and in the most derived Termitidae, the rumen was later re-internalized with the use of symbiotic bacteria as a base for cellulose digestion [53]. It was suggested that the origin of the fungal comb in a *Macrotermes* ancestor was the result of a primitive carton nest structure being invaded by *Termitomyces* [3]. Despite the presence of a carton material in *Coptotermes*, this genus, like all lower termites, never lost its protozoan endosymbionts, and apparently does not rely on an external rumen for nutritional purposes [54]. However, in the absence of the cultivation of a mutualistic fungus in *Coptotermes* nests, this carton material still represents a niche opportunity for various microorganisms [5,36] and we here showed that some of these microorganisms can provide a non-nutritional benefit to the termites in the form of an exogenous source of protection against diseases.

While our study was limited to a bioassay with a particular *Streptomyces* isolate (#2338), the presence of various *Streptomyces* strains and other Actinobacteria with a range of antimicrobial activity in all sampled termite nests, implies that multiple strains may be involved in such beneficial associations. For example, our *in vitro* result with *Streptomyces* #2345 does not fully exclude the potential survival benefit for the termites by a mechanism of exclusion competition *in vivo*. In addition, many other isolates should be tested individually or in association with other isolates (to explore eventual synergistic activity among isolates) to quantify their role in an active or potentially passive protection of termites against entomopathogens. This raises questions about the evolutionary traits of such an association in *Coptotermes*, considering the potential facultative nature of such symbiosis [55].

In attine ants, the mutualism with *Pseudonocardia* is a well-supported case of coevolution [29,56,57] with an adaptation of the ant cuticular structures to host the Actinobacteria biofilm [58,59]. However, recent studies [60–64] have suggested a potential recruitment of mutualists as a dynamic association, as an alternative to strict coevolution through obligatory vertical transmission. In the case of *C. formosanus*, a soil dwelling social insect also in recurrent contact with soil microorganisms during foraging, it can easily spread newly acquired microbes throughout the gallery system. This would result into a potentially dynamic symbiont turnover, as termites do not possess a specialized structure like attine ants [59]. In our study, *Streptomyces* isolates with identical 16S ribotype and similar antimicrobial activity to *Streptomyces* #2338 were present in all five *C. formosanus* nests surveyed. In addition, this 16S ribotype was also identical to many isolates from soil samples when compared to sequences in GenBank. However, the five carton materials evaluated were all sampled within Broward County, FL, USA and *C. formosanus* is an invasive species in southeastern United States that went through a strong bottleneck effect [65]. In addition, it is important to mention that the resolution of 16S gene may not provide sufficient evidence to confirm the soil recruitment hypothesis [22,56]. Therefore, it has yet to be established if the mutualistic association is the result of the recruitment of specific *Streptomyces* from local surrounding soils, if it is owing to vertical transmission by inheritance from the native distribution of the species, or a combination of both acquisition models [60,64].

While our current results may suggest that an assemblage of Actinobacteria, essentially originating from a community of free-living *Streptomyces*, invaded the termite tunnel system owing to the nutritional nature of the termite carton material, it remains to be confirmed. On-going surveys of the Actinobacteria community associated with the termite carton nest will provide valuable information: (i) a survey of the Actinobacteria community of termite nests from the current worldwide *C. formosanus* distribution will provide an overview of the Actinobacteria commonly associated with *C. formosanus* and determine whether this association can be maintained as a stable mutualism, (ii) the profiling of the Actinobacteria community from the surrounding soil of these termite nests will provide additional information that will help us refining the understanding of the mode of acquisition of this association and (iii) the sampling of the nest material, the gut content and the culticular microbial load of individuals from two generations of *C. formosanus* will cast light on the importance of both vertical and horizontal transmission of known isolates. Finally, the screening of the antimicrobial activity of all obtained isolates will provide unique information on their role in *C. formosanus* extended disease resistance, and could provide an alternative model for the study of evolution of defence mutualism in insects.
